# Supplementary material for: Global distribution patterns of marine nitrogen-fixers by imaging and molecular methods
Source: Nat Commun. 2021 Jul 6;12:4160. doi: 10.1038/s41467-021-24299-y (PMC8260585; doi:10.1038/s41467-021-24299-y)
Supplement: Supplementary file 1 — Supplementary Information [file 41467_2021_24299_MOESM1_ESM.pdf]

Supplementary Information

Global distribution patterns of marine nitrogen-fixers by imaging and molecular methods

Pierella Karlusich, et al.

Table of contents

Supplementary Figure S1: Comparison between the databases of diazotroph distribution and the *Tara* Oceans expeditions.

Supplementary Figure S2: Samples and methods used in this study.

Supplementary Figure S3: Biogeography of diatom-diazotroph associations (DDAs) in surface waters.

Supplementary Figure S4: Biogeography of *Trichodesmium* aggregates in surface waters.

Supplementary Figure S5: Abundance and distribution of free filaments of *Richelia*/*Calothrix* in surface waters by quantification of high-throughput confocal microscopy images in samples from size fraction 20-180 µm.

Supplementary Figure S6: *Trichodesmium* aggregation under different nutrient conditions.

Supplementary Figure S7: Putative spheroid bodies in *Tara* Oceans samples.

Supplementary Figure S8: Diazotroph community based on metagenomes from size-fractionated samples derived from deep-chlorophyll maxima.

Supplementary Figure S9: Diazotroph community based on metagenomes from size-fractionated samples from mesopelagic depths.

Supplementary Figure S10: Clusters of diazotroph communities in each size fraction based on metagenomes from surface samples.

Supplementary Figure S11: Clusters of diazotroph communities based on metagenomes from size-fractionated surface samples.

Supplementary Figure S12: Distribution of potential ultrasmall diazotrophs across metagenomes obtained in different size-fractionated samples.

Supplementary Figure S13: Description of the strategy used to discriminate microbial populations by flow cytometry from representative *Tara* Oceans stations.

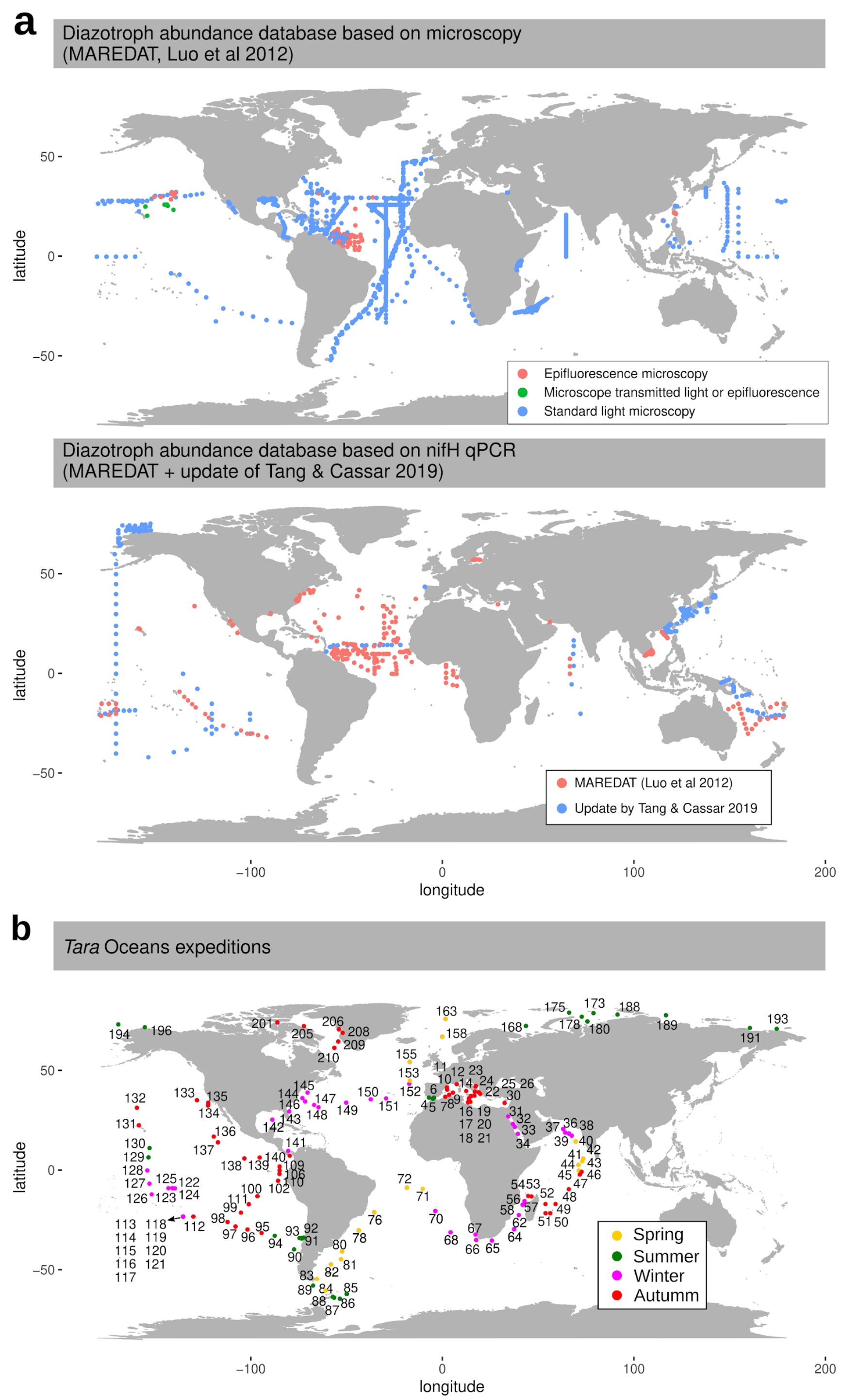

Supplementary Figure S1: Comparison between the databases of diazotroph distribution and the *Tara* Oceans expeditions. (**a**) The MARine Ecosystem DATa (MAREDAT) includes a database for microscopy counts (upper map) and for quantitative PCR targeting the *nifH* gene (lower map). The microscopy only covers *Trichodesmium* and diatom-diazotroph associations and it is a compilation of 44 different publications between 1966 and 2011 (Luo et al. 2012 *Earth Syst. Sci. Data* 4:47–73). The *nifH* dataset includes *Trichodesmium*, diatom-diazotroph associations, UCYN-A, *Crocosphaera* (UCYN-B) and UCYN-C and it is the result of 19 publications between 2005 and 2011 (red points; Luo et al. 2012 *Earth Syst. Sci. Data* 4:47–73). This later dataset has been recently updated (Tang and Cassar 2019 *Geophys. Res. Lett.* 46:12258–12269) with measurements from 17 new publications between 2012 and 2018 (blue points). (**b**) Sampling route of the *Tara* Oceans expeditions (2009-2013), showing station labels and sampling season.

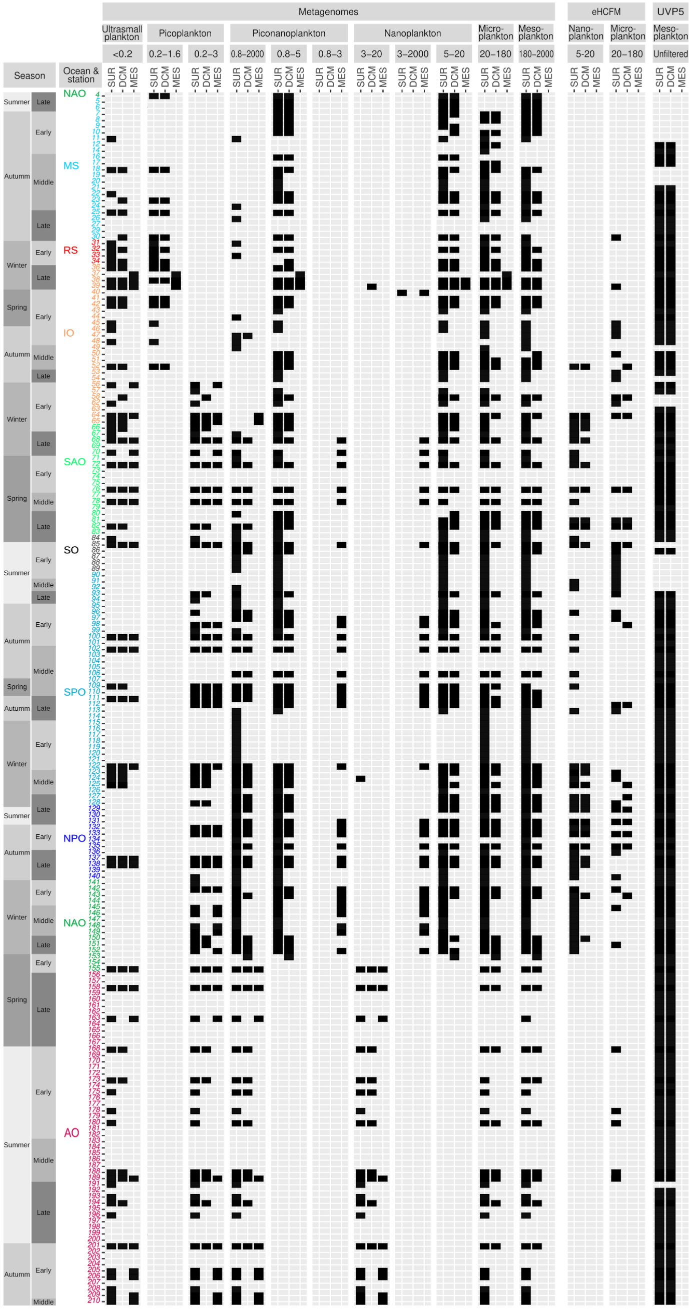

Supplementary Figure S2: Samples and methods used in this study. The current analysis of global diversity and abundance of diazotrophs was carried out across 197 *Tara* Oceans stations where samples where taken for metagenomic sequencing and/or for environmental High Content Fluorescence Microscopy (eHCFM) and/or images were taken *in situ* using an Underwater Vision Profiler 5 (UVP5). The analyzed samples are indicated as filled boxes. A complete sampling station consisted of collecting plankton from three distinct depth layers: surface (SUR), deep chlorophyll maximum (DCM), and mesopelagic (MES). The data from the bottom of the mixed layer was collected when no deep chlorophyll maximum was observed (stations TARA_123, TARA_124, TARA_125, TARA_152 and TARA_153). Plankton communities from SUR and DCM were fractionated into six main size classes: ultrasmall plankton (<0.22 μm), picoplankton (0.2 to 1.6 μm or 0.2 to 3 μm), piconanoplankton (0.8 to 5 μm or 0.8 to 2000 μm), nanoplankton (5 to 20 μm or 3 to 20 μm), microplankton (20 to 180 μm), and mesoplankton (180 to 2000 μm). For MES samples, size fractions were more heterogeneous (<0.22 μm, 0.2 to 1.6 μm, 0.2 to 3 μm, 0.8 to 3 μm, 0.8 to 5 μm, 0.8 to 200 μm, 0.8 to 2000 μm, 3-20 μm, 3-2000 μm, 5-20 μm). Season and moment of the season (early, middle, late) are displayed to the left of the panel. Station labels are coloured according to the ocean region: IO, Indian Ocean; MS, Mediterranean Sea; NAO, North Atlantic Ocean; RS, Red Sea; SAO, South Atlantic Ocean; SO, Southern Ocean; SPO, South Pacific Ocean.

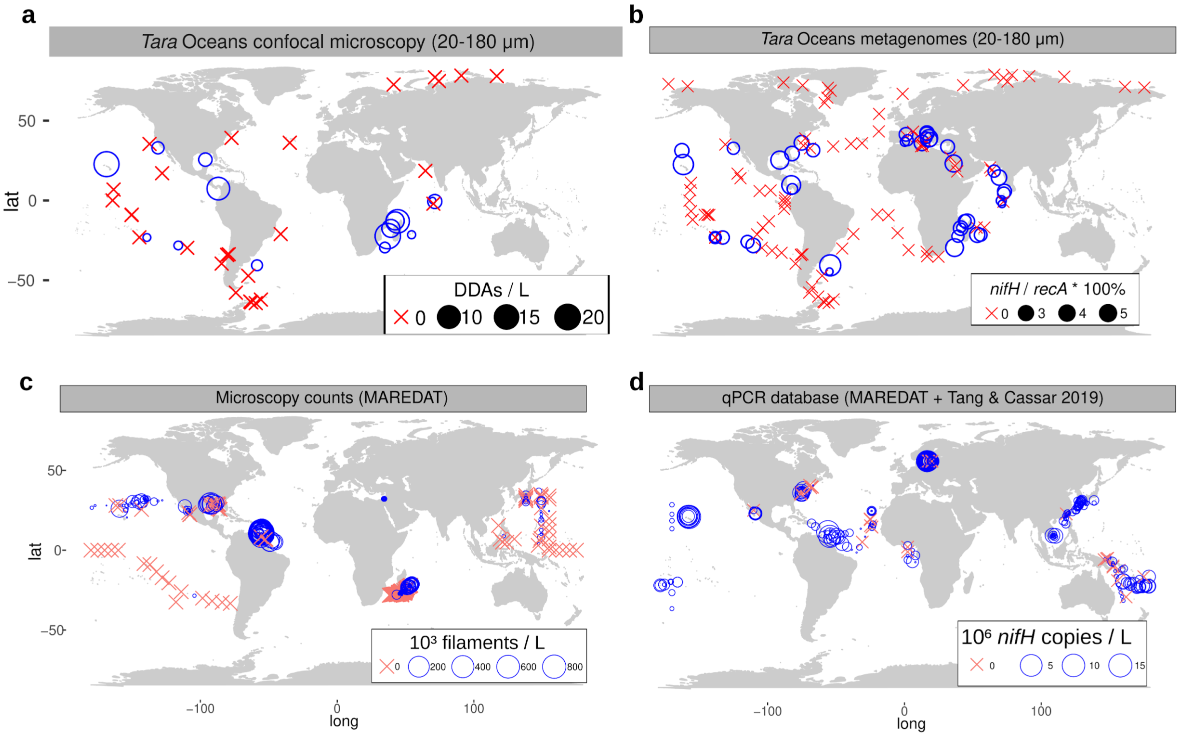

Supplementary Figure S3: Biogeography of diatom-diazotroph associations (DDAs) in surface waters. (**a-b**) Abundance across the *Tara* Oceans transect based on quantification of high-throughput confocal microscopy determinations (a) and from metagenomic read abundance of *nifH* gene (b). (**c-d**) Abundance in the MARine Ecosystem DATa (MAREDAT) database for microscopy counts (c) and for quantitative PCR targeting the *nifH* gene (d). This latter includes the recent compilation update by Tang and Cassar 2019. Bubble size varies according to the corresponding concentration, while crosses indicate their absence. Source data are provided as a Source Data file.

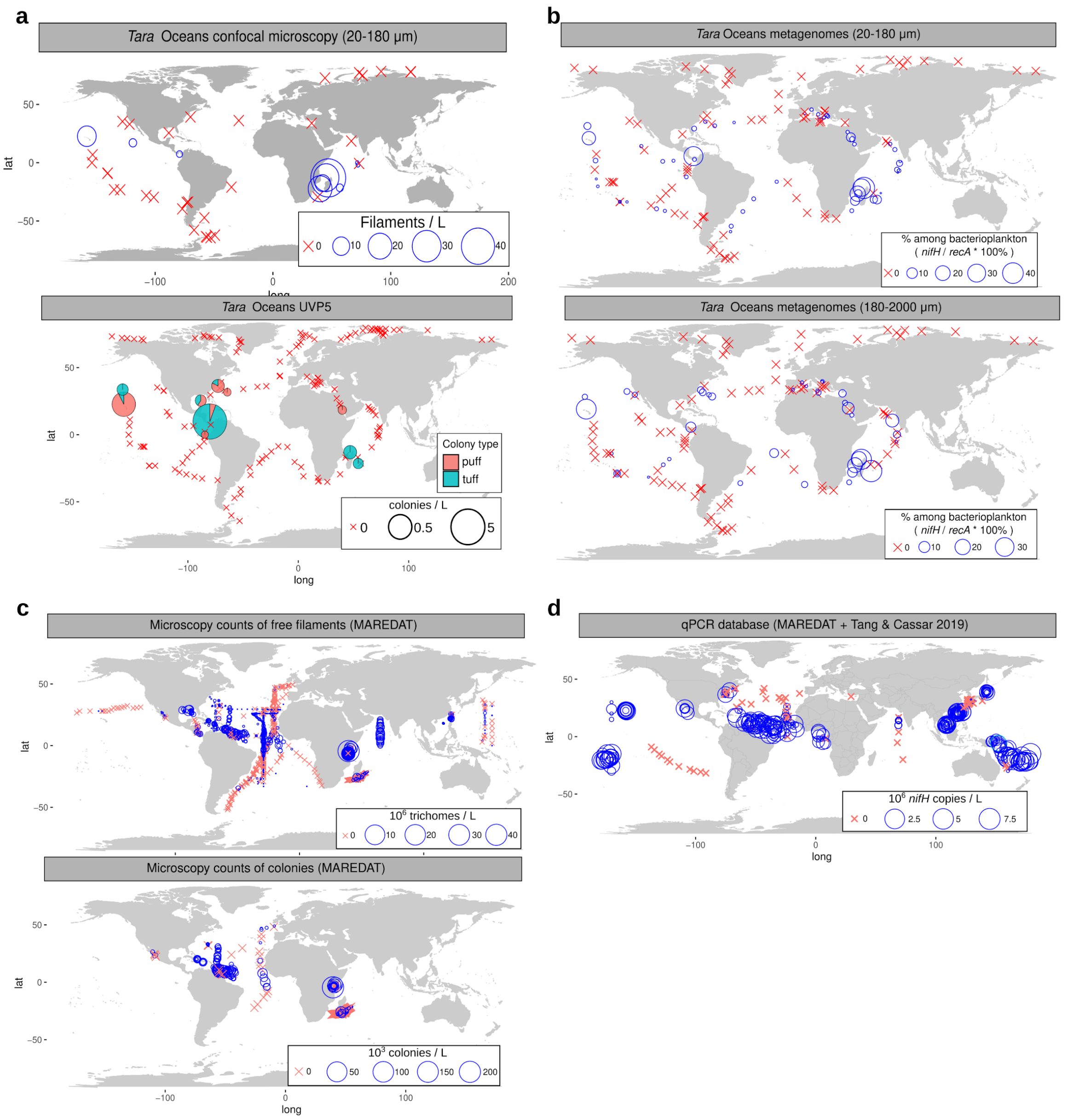

Supplementary Figure S4: Biogeography of *Trichodesmium* aggregates in surface waters. (**a**) Abundance across the *Tara* Oceans transect of free-filaments by high-throughput confocal microscopy in 20-180-μm size-fractionated samples (upper map) and colonies by Underwater Vision Profiler 5 (lower map). (**b**) Abundance across the *Tara* Oceans transect based on the metagenomic read abundance of the *nifH* marked gene in 20-180-μm and 180-2000-μm size-fractionated samples. (**c-d**) Abundance in the MARine Ecosystem DATa (MAREDAT) database for microscopy counts of free-filaments (c; upper map) and colonies (c; lower map) and for quantitative PCR targeting the *nifH* gene (d). This latter includes the recent compilation update by Tang and Cassar 2019. Bubble size varies according to the corresponding concentration, while crosses indicate their absence. Source data are provided as a Source Data file.

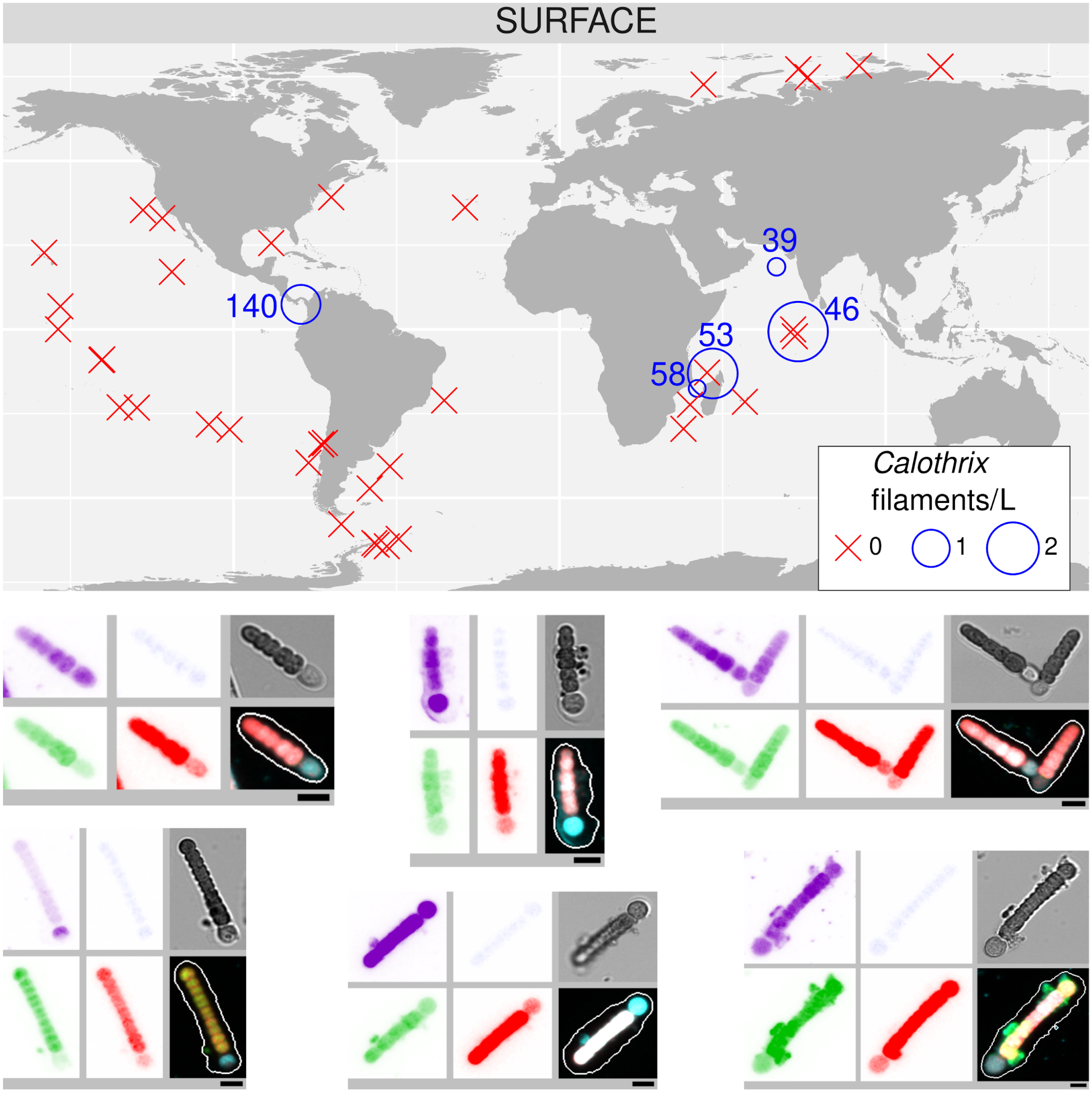

Supplementary Figure S5: Abundance and distribution of free filaments of *Richelia*/*Calothrix* in surface waters by quantification of high-throughput confocal microscopy images in samples from size fraction 20-180 µm. Maps show the biogeographical distribution. Bubble size varies according to the corresponding filament concentration, while red crosses indicate their absence. Examples of images are shown. From up left to bottom right, the displayed channels for each micrograph correspond to cell surface (cyan, AlexaFluor 546 dye), DNA (blue, Hoechst dye), cellular membranes (green, DiOC6 dye), chlorophyll autofluorescence (red), the bright field, and the merged channels. The size bar at the bottom left of each microscopy image corresponds to 2.5 µm. These micrographs are representatives of 20 images of the same annotation. Source data are provided as a Source Data file.

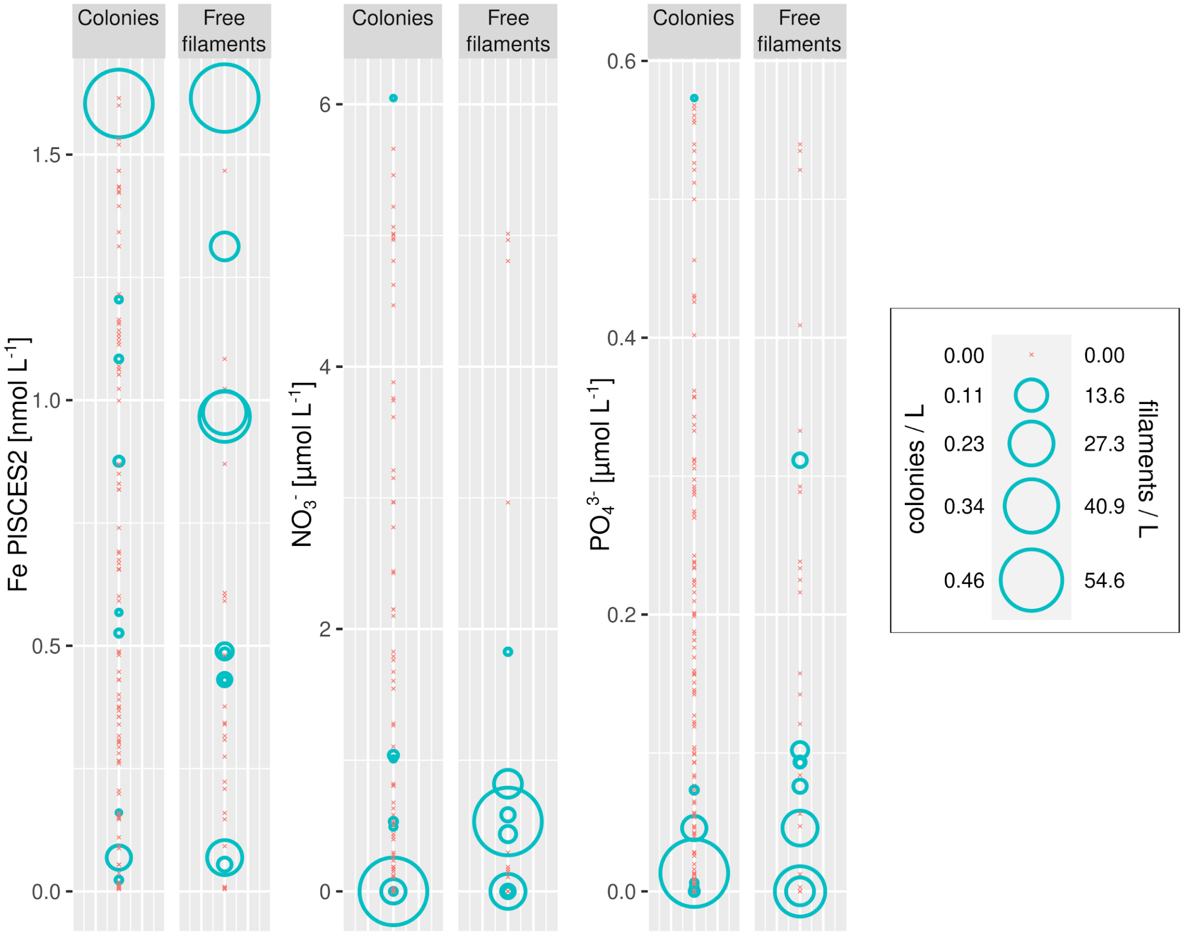

Supplementary Figure S6: *Trichodesmium* aggregation under different nutrient conditions. Bubble size varies according to the corresponding abundance of free filaments or colonies, while crosses indicate their absence. Free filaments were quantified by high-throughput confocal microscopy in samples from the 20-180 µm size fraction, while colonies were quantified using *in situ* images from the Underwater Vision Profiler 5 (UVP5). Source data are provided as a Source Data file.

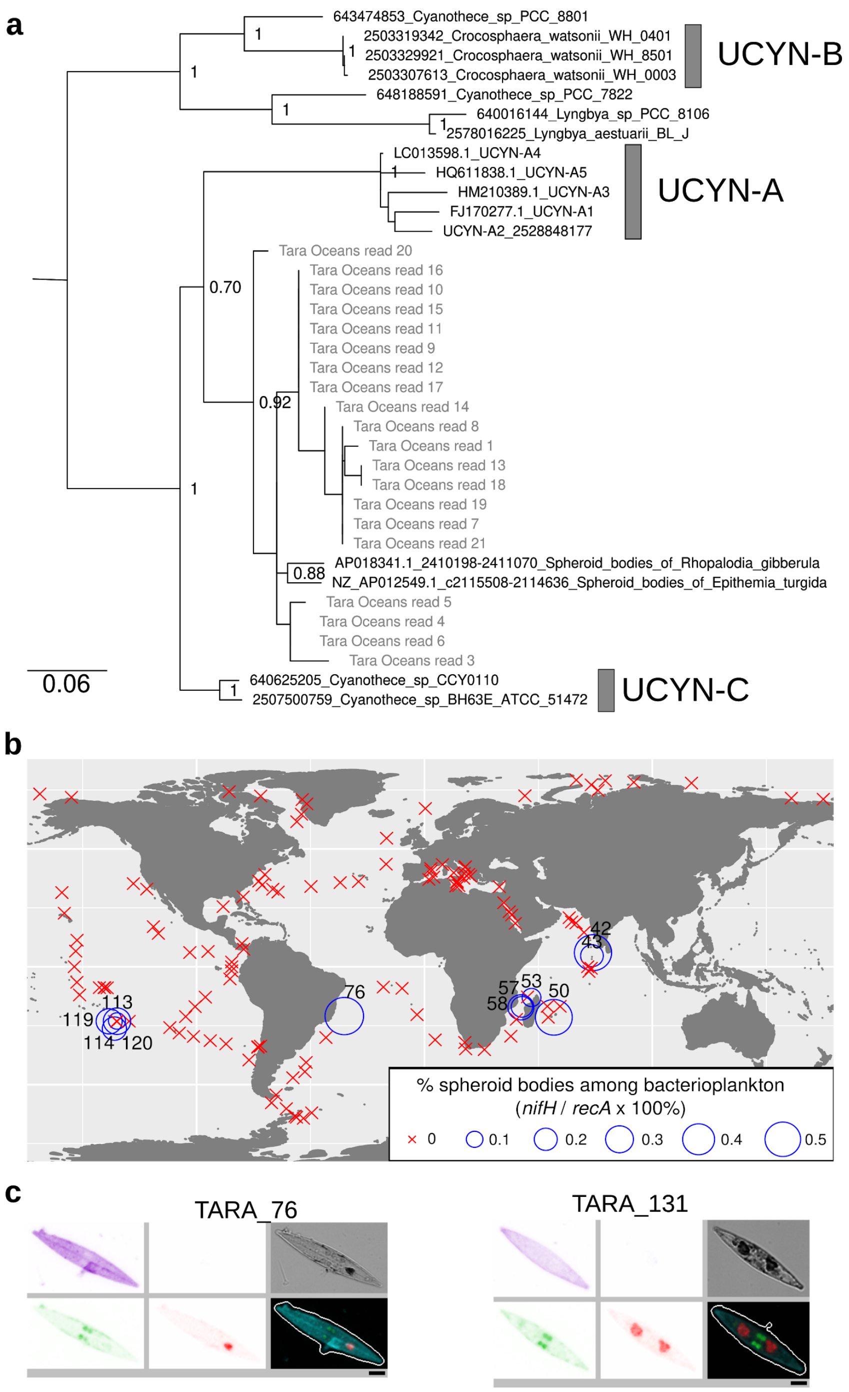

Supplementary Figure S7: Putative spheroid bodies in *Tara* Oceans samples. (**a**) Phylogeny of metagenomic reads with sequence similarity to the *nifH* gene from spheroid bodies. NCBI or IMG accession numbers of reference nucleotide sequences and the species names are indicated in the tip labels. The aLRT values are shown for the main clades. (**b**) Biogeography in surface waters of 20-180 µm size fractionated samples. The bubble size varies according to the percentage of reads of potential spheroid bodies, while crosses indicate absence (i.e., no detection of *nifH* reads). Station labels with read detection are indicated. (**c**) Images of pennate diatoms containing round granules that lack chlorophyll autofluorescence that were observed in the same samples where putative metagenomic sequences from spheroid-bodies were detected. From up left to bottom right, the displayed channels for each micrograph correspond to cell surface (cyan, AlexaFluor 546 dye), DNA (blue, Hoechst dye), cellular membranes (green, DiOC6 dye), chlorophyll autofluorescence (red), the bright field, and the merged channels. The size bar at the bottom left of each microscopy image corresponds to 2.5 µm. These micrographs are representatives of 12 images for the same annotation. Source data are provided as a Source Data file.

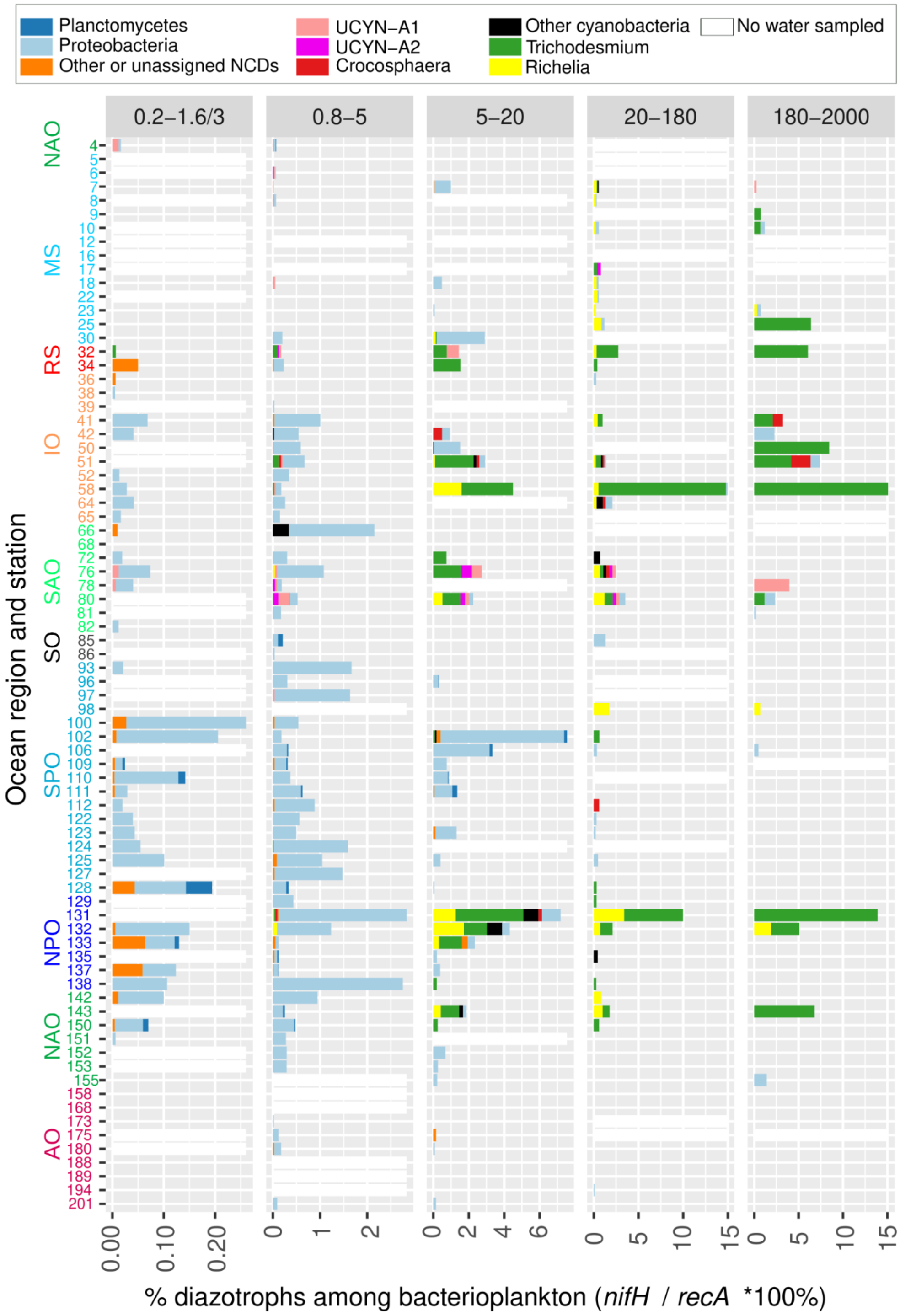

Supplementary Figure S8: Diazotroph community based on metagenomes from size-fractionated samples derived from deep-chlorophyll maxima. The percentage of diazotrophs in the bacterioplankton community was estimated by the ratio of metagenomic read abundance between the marker genes *nifH* and *recA*. The bar color code shows the taxonomic annotation, and the absence of water sample is indicated by a white bar. The Y axis shows the *Tara* Oceans stations and the ocean regions. Abbreviations: MS, Mediterranean Sea; IO, Indian Ocean; SAO, South Atlantic Ocean; SO, Southern Ocean; SPO, South Pacific Ocean; NPO, North Pacific Ocean; NAO, North Atlantic Ocean; AO, Arctic Ocean. The equivalent figure showing the surface layer is shown in Figure 7 (note the differences in scales between both figures, showing the higher relative abundance of diazotrophs in the surface layer). The data from the bottom of the mixed layer is displayed when no deep chlorophyll maximum was observed (stations TARA_123, TARA_124, TARA_125, TARA_152 and TARA_153). Source data are provided as a Source Data file.

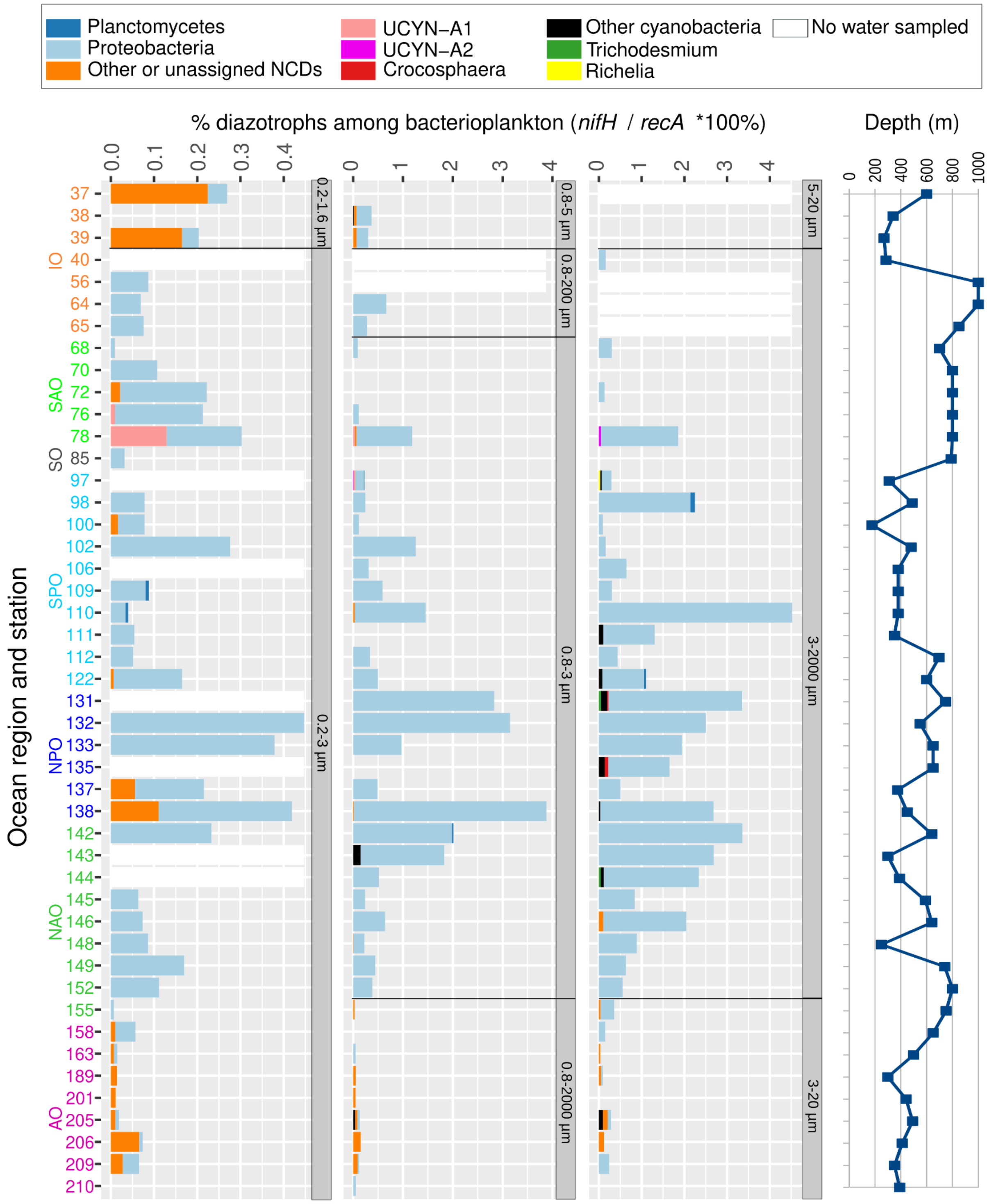

Supplementary Figure S9: Diazotroph community based on metagenomes from size-fractionated samples from mesopelagic depths. The percentage of diazotrophs in the bacterioplankton community was estimated by the ratio of metagenomic read abundance between the marker genes *nifH* and *recA*. The bar color code shows the taxonomic annotation, and the absence of water sample is indicated by a white bar. Size fractions are also indicated (they are more heterogeneous than those from surface and deep chlorophyll maximum samples). The Y axis shows the *Tara* Oceans stations and the ocean regions. Abbreviations: MS, Mediterranean Sea; IO, Indian Ocean; SAO, South Atlantic Ocean; SO, Southern Ocean; SPO, South Pacific Ocean; NPO, North Pacific Ocean; NAO, North Atlantic Ocean; AO, Arctic Ocean. Sampling depth is indicated in the right panel. Source data are provided as a Source Data file.

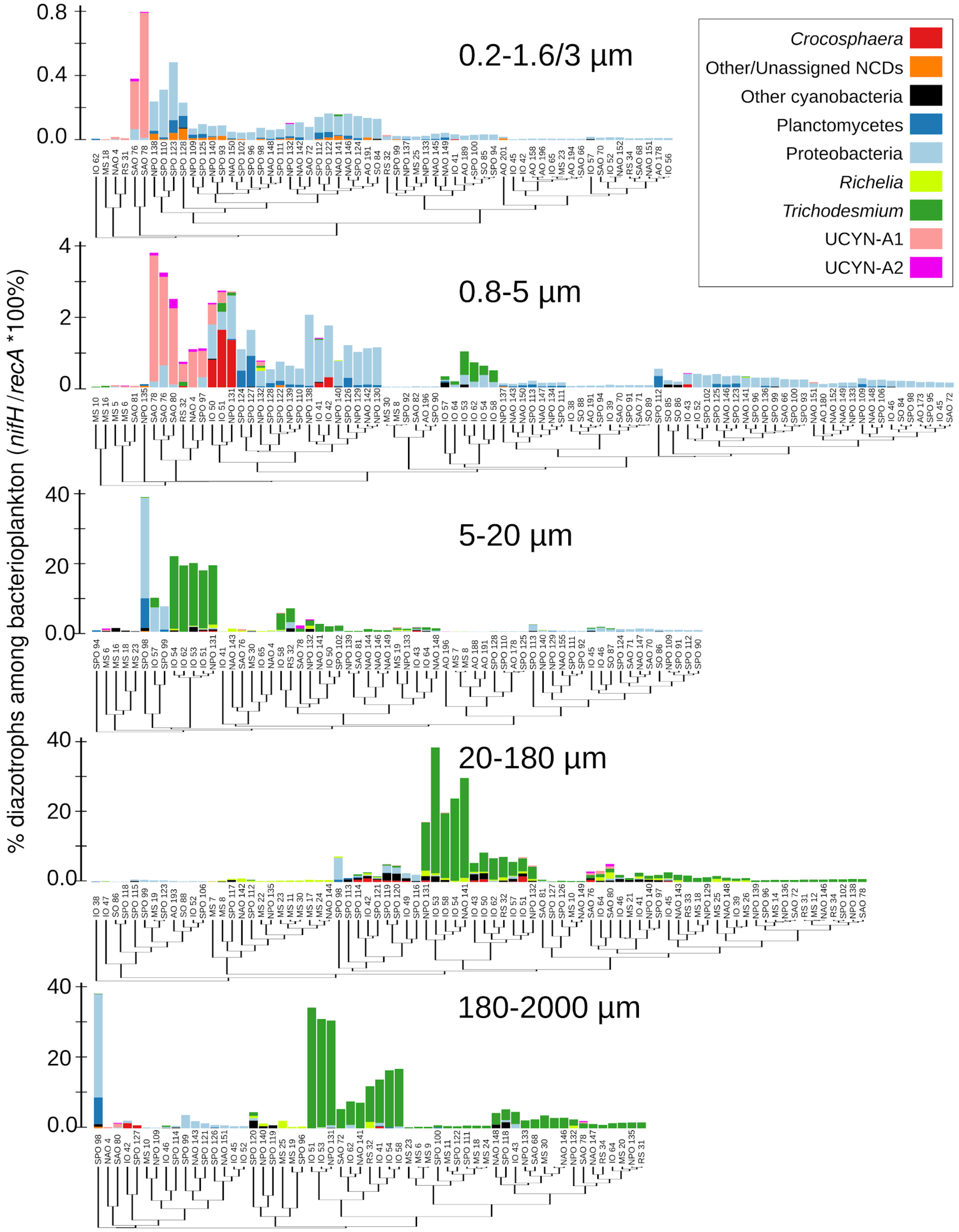

Supplementary Figure S10: Clusters of diazotroph communities in each size fraction based on metagenomes from surface samples. For each size fraction, the samples are sorted by similarity using hierarchical clustering (Bray–Curtis distance) and the corresponding diazotroph relative abundances are displayed as bar plots, with the color code according to the taxonomic annotation. The percentage of diazotrophs in the bacterioplankton community was estimated by the ratio of metagenomic read abundance between the marker genes *nifH* and *recA*. The dendrogram tip labels show the *Tara* Oceans stations and the ocean regions. Abbreviations: MS, Mediterranean Sea; IO, Indian Ocean; SAO, South Atlantic Ocean; SO, Southern Ocean; SPO, South Pacific Ocean; NPO,

North Pacific Ocean; NAO, North Atlantic Ocean; AO, Arctic Ocean. Source data are provided as a Source Data file.

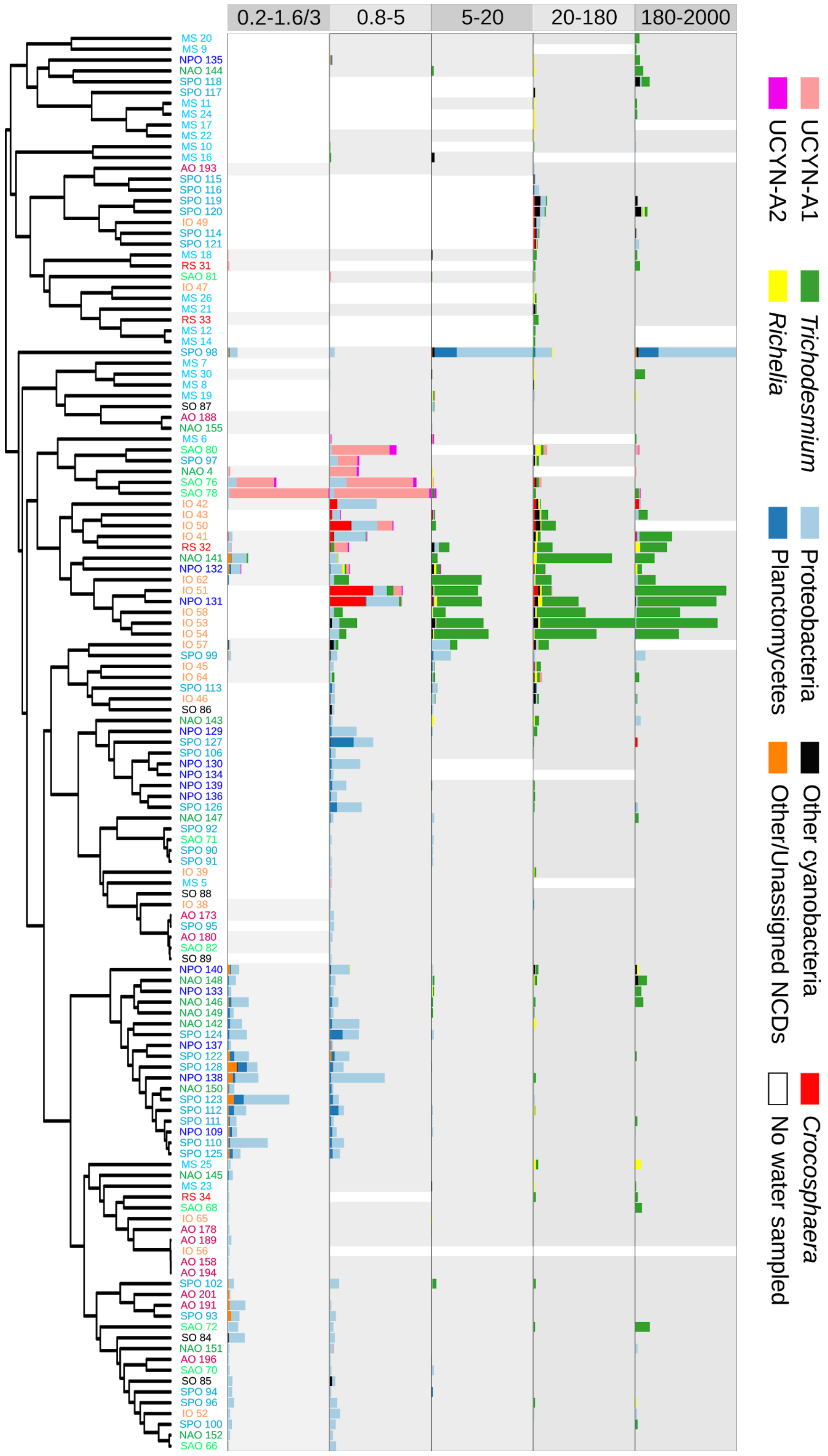

Supplementary Figure S11: Clusters of diazotroph communities based on metagenomes from size-fractionated surface samples. For each size fraction, the samples are sorted by similarity using hierarchical clustering (Bray–Curtis distance) and the corresponding diazotroph relative abundances are displayed as bar plots, with the color code according to the taxonomic annotation. The percentage of diazotrophs in the bacterioplankton community was estimated by the ratio of metagenomic read abundance between the marker genes *nifH* and *recA*. The dendrogram tip labels show the *Tara* Oceans stations and the ocean regions. Abbreviations: MS, Mediterranean Sea; IO, Indian Ocean; SAO, South Atlantic Ocean; SO, Southern Ocean; SPO, South Pacific Ocean; NPO, North Pacific

Ocean; NAO, North Atlantic Ocean; AO, Arctic Ocean. Source data are provided as a Source Data file.

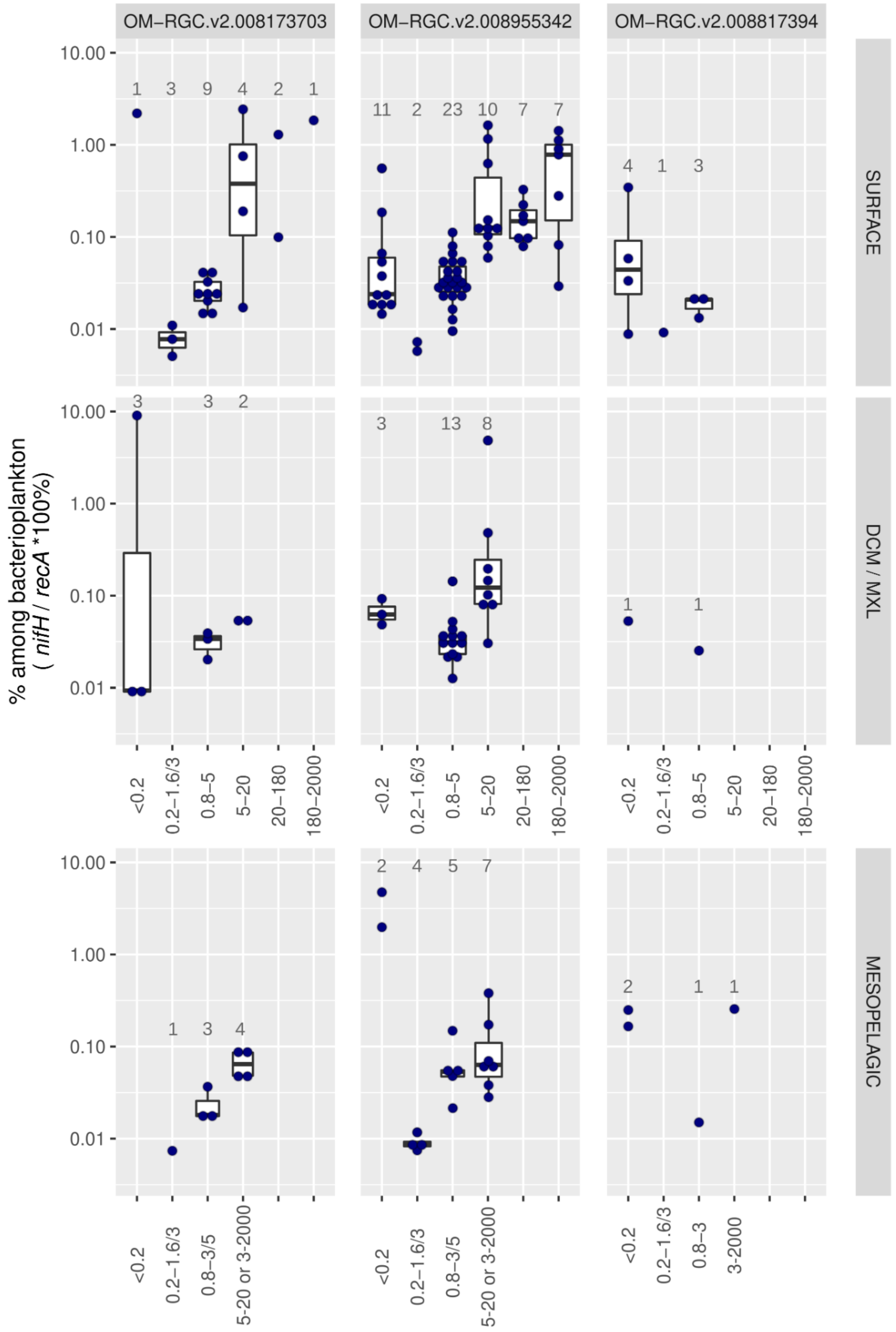

Supplementary Figure S12: Distribution of potential ultrasmall diazotrophs across metagenomes obtained in different size-fractionated samples. For each taxon, the percentage in the bacterioplankton community is estimated by the ratio of metagenomic read abundance between the marker genes *nifH* and *recA*. The 'OM-RGC.v2' prefix indicates the *nifH* sequences assembled from the metagenomes of <0.22 μm size fraction (Salazar al. 2019 *Cell* 179:1068–1083.e21). Boxplots depict the 25–75% quantile range of the dataset without zeros (the corresponding biologically independent seawater samples are indicated in the plot), with the center line depicting the median

(50% quantile); whiskers encompass data points within 1.5x the interquartile range. Source data are provided as a Source Data file.

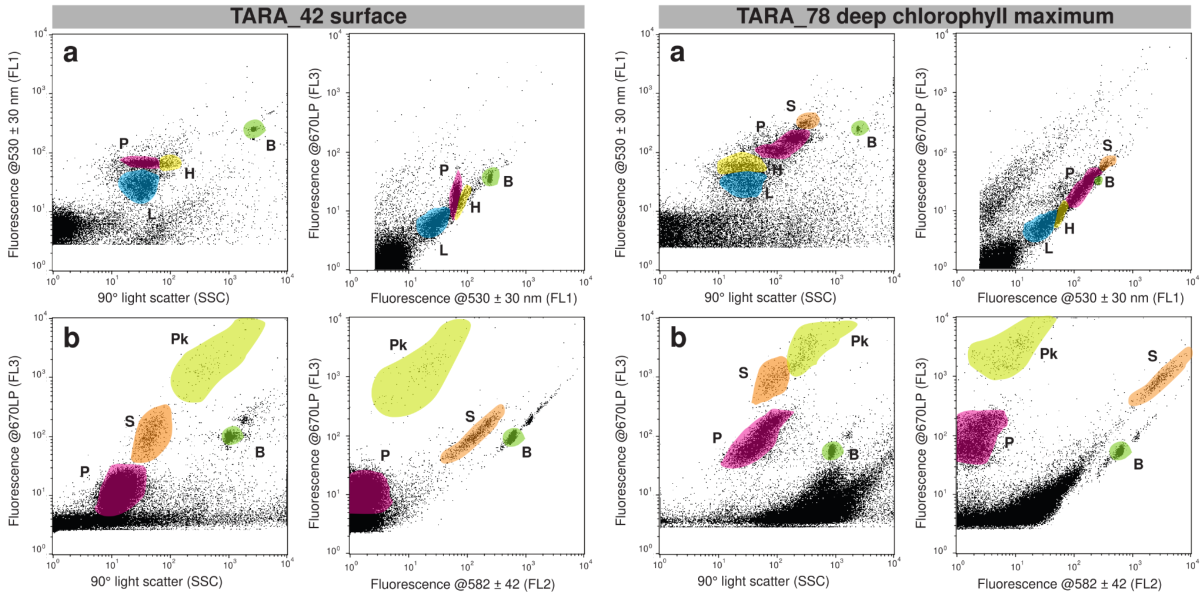

Supplementary Figure S13: Description of the strategy used to discriminate microbial populations by flow cytometry from representative *Tara* Oceans stations. (**a**) Each sample was stained with SybrGreen 1 and a combination of side scatter (SSC) as well as nucleic-acid derived SybrGreen green fluorescence (FL1) and chlorophyll red autofluorescence (FL3) were used to discriminate two populations of heterotrophic prokaryotes, high-nucleic acid containing (HNA, H), and low nucleic-acid containing (LNA, L), as well as *Prochlorococcus* (P), and *Synechococcus* (S), although this last population not in all samples. (**b**) Unstained samples could discriminate autofluorescent populations based on size (using the surrogate side scatter), chlorophyll content (red fluorescence, FL3), and phycoerythrin content (orange fluorescence, FL2). This method allows to discriminate *Prochlorococcus* (P), from *Synechococcus* (S), and small photosynthetic picoeukaryotes (Pk). In some samples a larger population of nanoeukaryotes is also visible. The examples correspond to surface waters of station TARA_42 (left) and deep chlorophyll maximum waters of station TARA_78 (right). Deeper populations have larger per cell chlorophyll and phycoerythrin contents as they adapt to the available light. Standard Polysciences 1 µm yellow-green beads (B) are always added as a reference and to allow comparison of fluorescence and scatter from sample to sample.
