# Supplementary material for: Global distribution patterns of marine nitrogen-fixers by imaging and molecular methods
Source: Nat Commun. 2021 Jul 6;12:4160. doi: 10.1038/s41467-021-24299-y (PMC8260585; doi:10.1038/s41467-021-24299-y)
Supplement: Supplementary file 3 — Description of Additional Supplementary Files [file 41467_2021_24299_MOESM3_ESM.docx]

**Description of Additional Supplementary Files**

Title: Supplementary Data 1

Description: Metagenomic read mapping against nifH and recA sequence catalogs

Title: Supplementary Data 2

Description: Polyploidy estimations in Trichodesmium and Richelia/Calothrix.

Title: Supplementary Data 3

Description: catalog of nifH sequences and homologous

Title: Supplementary Data 4

Description: Compilation of recA sequences as well as homologous used as outgroups.

Title: Supplementary data 5

Description: Initial (manually-curated) training set for diatom-diazotroph associations and free filaments of Trichodesmium.

Title: Supplementary Data 6

Description: Contextual data for the Tara Oceans samples used in the current work.
