# Supplementary material for: Global distribution patterns of marine nitrogen-fixers by imaging and molecular methods
Source: Nat Commun. 2021 Jul 6;12:4160. doi: 10.1038/s41467-021-24299-y (PMC8260585; doi:10.1038/s41467-021-24299-y)
Supplement: Supplementary file 10 — Reporting Summary [file 41467_2021_24299_MOESM10_ESM.pdf]

nature research

Corresponding author(s): Rachel A Foster, Chris Bowler

Last updated by author(s): May 12, 2021

Reporting Summary

Nature Research wishes to improve the reproducibility of the work that we publish. This form provides structure for consistency and transparency in reporting. For further information on Nature Research policies, see our Editorial Policies and the Editorial Policy Checklist.

Statistics

For all statistical analyses, confirm that the following items are present in the figure legend, table legend, main text, or Methods section.

n/a	Confirmed	
☐	☒	The exact sample size (*n*) for each experimental group/condition, given as a discrete number and unit of measurement
☐	☒	A statement on whether measurements were taken from distinct samples or whether the same sample was measured repeatedly
☐	☒	The statistical test(s) used AND whether they are one- or two-sided<br>*Only common tests should be described solely by name; describe more complex techniques in the Methods section.*
☒	☐	A description of all covariates tested
☒	☐	A description of any assumptions or corrections, such as tests of normality and adjustment for multiple comparisons
☐	☒	A full description of the statistical parameters including central tendency (e.g. means) or other basic estimates (e.g. regression coefficient) AND variation (e.g. standard deviation) or associated estimates of uncertainty (e.g. confidence intervals)
☐	☒	For null hypothesis testing, the test statistic (e.g. *F*, *t*, *r*) with confidence intervals, effect sizes, degrees of freedom and *P* value noted<br>*Give P values as exact values whenever suitable.*
☒	☐	For Bayesian analysis, information on the choice of priors and Markov chain Monte Carlo settings
☐	☒	For hierarchical and complex designs, identification of the appropriate level for tests and full reporting of outcomes
☐	☒	Estimates of effect sizes (e.g. Cohen's *d*, Pearson's *r*), indicating how they were calculated

Our web collection on statistics for biologists contains articles on many of the points above.

Software and code

Policy information about availability of computer code

Data collection	No software was used for data collection as no samples were taken for this specific study and we used data sources that are open and publicly available. The software that was previously used for data collection to generate these datasets includes Paint-A-Gate software (Becton and Dickinson) for flow cytometry and the source code of the computational pipeline for environmental high content fluorescence microscopy data processing available at https://git.embl.de/coelho/eHCFM.
Data analysis	The following workflow applies to the Image analyses: search, annotation and curation of e-HCFM and UVP5 images were carried out using the open website resource Ecotaxa version 2 (https://ecotaxa.obs-vlfr.fr/).<br>The following applies to the sequences based analyses. The searches for nifH and recA reference sequences and their homologs in reference databases was carried out using HMMer version 3.2.1 (http://hmmer.org/). Metagenome read recruitments were carried out with bwa version 0.7.4 (http://bio-bwa.sourceforge.net/). Phylogenetic analysis of metagenomic reads of interest was carried out using the alignment tools MAFFT version 6 (https://mafft.cbrc.jp/alignment/software/) and TranslatorX version 1 (http://translatorx.co.uk/), and the phylogenetic tool PhyML version 3.0 (http://www.atgc-montpellier.fr/phyml/usersguide.php).<br>Plots and general analyses were carried out using R version 3.6.0 (http://www.r-project.org/) using the libraries ggplot2 version 3.2.1 (https://cran.r-project.org/web/packages/ggplot2/index.html), scatterpie version 0.1.5 (https://cran.r-project.org/web/packages/scatterpie/vignettes/scatterpie.html), treemap version 2.4.2 (https://cran.r-project.org/web/packages/treemap/index.html), vegan version 2.5.5 (https://cran.r-project.org/web/packages/vegan/index.html), and stats version 3.6.0 (https://cran.r-project.org/web/packages/STAT/index.html).

For manuscripts utilizing custom algorithms or software that are central to the research but not yet described in published literature, software must be made available to editors and reviewers. We strongly encourage code deposition in a community repository (e.g. GitHub). See the Nature Research guidelines for submitting code & software for further information.

Data

Policy information about availability of data

All manuscripts must include a data availability statement. This statement should provide the following information, where applicable:

- Accession codes, unique identifiers, or web links for publicly available datasets
- A list of figures that have associated raw data
- A description of any restrictions on data availability

For simplicity we have divided the data and its availability as follows:
1. HYDROGRAPHIC DATA: in situ variables which are measured a the time of sampling including sampling location (ocean region, latitude, longitude, depth) and environmental variables (temperature, nutrient concentrations), was retrieved from Pangaea: https://doi.org/10.1594/PANGAEA.875582
2. FLOW CYTOMETRY: The last version of the flow cytometry Tara Oceans dataset published by Ibarbalz et al. 2019 Cell 179:1084-1097 was used. Data is open and publicly available from http://dx.doi.org/10.17632/p9r9wttjkm.2
3. e-HCFM images from samples of size fractions 5-20 µm and 20-180 µm and their metadata were retrieved from Ecotaxa: https://ecotaxa.obs-vlfr.fr/prj/3365 and https://ecotaxa.obs-vlfr.fr/prj/2274 , respectively.
4. UVP5 images and their metadata were retrieved from Ecotaxa: https://ecotaxa.obs-vlfr.fr/prj/579
5. The e-HCFM and UVP5 images that were annotated and analysed in the current work (i.e., predicted and annotated as diazotrophs) were submitted to the EMBL-EBI repository BioStudies (www.ebi.ac.uk/biostudies) under accession S-BSST529.
6. Tara Oceans metagenomes are archived at ENA under the accession numbers: PRJEB1787, PRJEB1788, PRJEB4352, PRJEB4419, PRJEB9691, PRJEB9740, PRJEB402 and PRJEB9742.
7. The nifH catalog consisted of the version April 2014 of the publicly available database curated and hosted at the marine microbial ecology Zehr Lab, University of California, Santa Cruz, CA USA (https://www.jzehrlab.com/nifh), and complemented with additional sequences from sequenced genomes (IMG, https://img.jgi.doe.gov/) and from different Tara Oceans datasets: OM-RGC-v2 (https://www.ocean-microbiome.org/) and assemblies (Supplementary Table 8 in Delmont et al 2018 Nat Microbiol 3:804–813; https://static-content.springer.com/esm/art%3A10.1038%2Fs41564-018-0176-9/MediaObjects/41564_2018_176_MOESM10_ESM.xlsx) and 10 sequenced clones from the current study (accession numbers MW590317-MW590326 at ENA; https://www.ebi.ac.uk/ena/browser/home).
8. The recA catalog was compiled from Integrated Microbial Genomes (IMG, https://img.jgi.doe.gov/); Marine Microbial Eukaryotic Transcriptome Sequencing Project (MMETSP; https://github.com/dib-lab/dib-MMETSP); OM-Reference Gene Catalog version 2 (OM-RGC-v2, https://www.ocean-microbiome.org/).

Field-specific reporting

Please select the one below that is the best fit for your research. If you are not sure, read the appropriate sections before making your selection.

☐ Life sciences ☐ Behavioural & social sciences ☒ Ecological, evolutionary & environmental sciences

For a reference copy of the document with all sections, see nature.com/documents/nr-reporting-summary-flat.pdf

Ecological, evolutionary & environmental sciences study design

All studies must disclose on these points even when the disclosure is negative.

Study description	We report the distribution and abundance patterns of marine nitrogen-fixers (diazotrophs) at different water layers (surface, deep chlorophyll maximum, mesopelagic) by mining metagenomes and imaging datasets generated by the Tara Oceans expeditions (2009-2013). The design type of Tara Oceans expeditions is observation design and global survey.
Research sample	No samples were taken for this study because we mined the datasets generated by the Tara Oceans expeditions (2009-2013) that are open and publicly available (see data availability statement in the current document). The datasets were generated from seawater samples collected at different depths and locations across the main ocean regions. The plankton were separated into discrete size fractions using a serial filtration system, and given the inverse logarithmic relationship between plankton size and abundance, higher seawater volumes were filtered for the larger size fractions (see 'Sampling strategy'). The targeted population of this paper corresponds to nitrogen-fixers (diazotrophs) and taking into account that diazotrophs are less abundant than sympatric populations and have a wide size variation, the Tara Oceans datasets were useful for a comprehensive perspective over a broad spectrum, which to date has been lacking. Thus, our work included the full biological and ecological complexity of diazotrophs: i.e., unicellular, colonial, particle associated, symbiotic, cyanobacteria and on cyanobacterial diazotrophs (NCDs). Examples of these groups are i) the colony-forming non-heterocystous Trichodesmium spp.; ii) the heterocystous cyanobacterial (Richelia intracellularis and Calothrix rhizosoleniae) symbionts of diatoms, iii) unicellular cyanobacteria (UCYN), including Candidatus Atelocyanobacterium thalassa (UCYN-A) and Crocosphaera watsonii (UCYN-B); iv) non cyanobacterial diazotrophs (NCDs) including species of Proteobacteria and Planctomycetes.
Sampling strategy	Sampling strategy for Tara Oceans included the sampling of three main water layers: surface (5 m depth), deep chlorophyll maximum (17–188 m), and mesopelagic (200–1000 m). Plankton samples were separated into discrete size fractions using a serial filtration system. Plankton communities from surface and deep chlorophyll maximum were fractionated into six main size classes: ultrasmall plankton (<0.22 µm), picoplankton (0.2 to 1.6 µm or 0.2 to 3 µm), piconanoplankton (0.8 to 5 µm or 0.8 to 2000 µm), nanoplankton (5 to 20 µm or 3 to 20 µm), microplankton (20 to 180 µm), and mesoplankton (180 to 2000 µm). For mesopelagic samples, size fractions were more heterogeneous (<0.22 µm, 0.2 to 1.6 µm, 0.2 to 3 µm, 0.8 to 3 µm, 0.8 to 5 µm, 0.8 to 200 µm, 0.8 to 2000 µm, 3-20 µm, 3-2000 µm, 5-20 µm). Given the inverse logarithmic relationship between plankton size and abundance (Belgrano et al 2002 Ecol. Lett. 5: 611–613), higher seawater volumes were filtered for the larger size fractions (10-105 L; see Table 1 and Figure 5 in Pesant et al. 2015 Sci Data 2:150023). Species richness estimates are important when designing sampling strategies and methodologies for biodiversity studies. Based on literature reports, it was estimated that the sampled seawater volumes of the Tara

	Oceans expedition would capture <50% of total richness for plankton in the 0.8–5 µm size fraction (Pesant et al. 2015 Sci Data 2:150023). Accordingly, one would need to filter thousands of litres of seawater in order to capture 75% of total richness for these groups. This is both impractical for most field campaigns and dependent on how one defines the current richness for these groups, i.e., the concept of species. In the higher size fractions (>5 µm), the sampling strategy appears to have captured 75-100% of species richness (Pesant et al. 2015 Sci Data 2:150023). It is important to note that data about plankton total richness is still very scarce in the literature, so that this assessment is only a first approximation.A suite of size-fractionated samples were taken and archived for later analyses, including those relevant to our current manuscript such as fixed samples for flow cytometry, DNA samples for sequencing and fixed samples for e-HCFM and are reported in the following: Colin et al. 2017 Elife 6: e26066, Alberti et al. 2017 Sci Data 4: 170093, Hingamp et al. 2013 ISME J 7.9: 1678-1695.
Data collection	This study used existing data sources. All data sources are open and publicly available (see data availability statement in the current document). Measurements of temperature were recorded at the time of sampling using the vertical profile sampling system (CTD-rosette) and Niskin bottles following the sampling package described in https://doi.pangaea.de/10.1594/PANGAEA.836319 and https://doi.pangaea.de/10.1594/PANGAEA.836321. Dissolved nutrients (NO3-, PO43-) were analyzed according to previous methods (Murphy & Riley 1962 Anal Chim Acta 27:31–36; Bendschneider & Robinson 1952 J Mar Res 11:87–96). Iron levels were derived from a global ocean biogeochemical model (Aumont et al 2015 Geosci Model Dev 8:2465–2513).<br>Nucleic acid samples were stored in liquid nitrogen on R/V Tara and were transferred on dry ice approximately every 6 weeks from a port of call to Frankfurt airport (Germany), from where they were subsequently shipped to Genoscope in Ivry (France) for DNA extraction and sequencing. a comprehensive description of the nucleic acid extraction and sequencing methods is described in Alberti et al. 2017 Sci Data 4: 1-20.<br>Regarding imaging, the e-HCFM method is based on an automated Leica SP8 TCS confocal laser scanning microscope that enables 3D multicolor imaging of cells (Colin et al., 2017 Elife 6: e26066). The instrument was developed at EMBL and analysed samples which were previously chemically fixed (1% paraformaldehyde and 0.25% glutaraldehyde) and stored at 4 °C until analysis. In the current work, we analysed the e-HCFM dataset from 5-20 and 20-180 µm size-fractionated samples. Another imaging dataset used images generated by an UVP5 mounted on the Rosette Vertical Sampling System. This system allows to illuminate precisely calibrated volumes of water and capture images at a rate of 5 to 20 images s−1 during the descent (Picheral et al. 2010 Limnol. Oceanogr 8: 462–473). The UVP5 was operated in situ and was designed to detect and count objects of >100 µm in length and to identify those of >600 µm in size.
Timing and spatial scale	From its departure in September 2009 to its return in December 2013, SV Tara sailed 140,000 km over a period of 38 months, systematically collecting more than 35,000 ocean water and plankton samples as well as environmental data at 210 stations across the Mediterranean Sea, Red Sea, Indian Ocean, South Atlantic Ocean, Southern Ocean, South Pacific Ocean, North Pacific Ocean, North Atlantic Ocean, and Arctic Ocean. The aim was to sample most of the biogeographic and biogeochemical provinces (Longhurst 1995 Prog. Oceanogr. 36:77–167) of the global ocean and to target well-defined mesoscale features such as gyres, eddies, currents, frontal zones, upwellings, hotspots of biodiversity, low pH or low oxygen zones (see for example, the study of the plankton community in Agulhas rings by Villar et al. 2015 Science 348:1261447, or in the Marquesas archipelago by Caputi et al. 2019 Global Biogeochem Cy 33:391–419), and to follow standardized protocols and logistics for sample collection, distribution and storage to facilitate comparative analysis. Real-time remote sensing and other data were leveraged to locate oceanographically interesting features (e.g., eddies, fronts, upwellings) and strengthen ecosystem comparisons (Karsenti et al. 2011 PLoS biol 9.10: e1001177.) A complete sampling station consisted of collecting plankton from three distinct environmental features, typically the surface water layer, the deep chlorophyll maximum layer, and mesopelagic zone. A sampling station lasted typically 48 hours, and the sequence of sampling deployments generally followed the same order (Pesant et al. 2015 Sci Data 2.1: 1-16). Metagenomic, imaging and hydrographic data were used in this study. The 1,326 metagenomes generated by the expedition are derived from 147 globally distributed stations and three different water layers: 745 metagenomes from surface, 382 from DCM (17–188 m) and 41 from the bottom of the mixed layer when no DCM was observed (25-140 m), and 158 from mesopelagic (200–1000 m). The eHCFM dataset analysed in the current work consists of: i) 75 samples of size-fraction 5-20 µm at 51 stations; ii) 61 samples of size fraction 20-180 µm at 48 different stations. The flow cytometry dataset consists of 212 samples collected at 137 stations. UVP5 measurements were taken in situ across 174 stations. See also Supplementary Figure S2.
Data exclusions	No data were excluded from this study.
Reproducibility	All measurements done by Tara Oceans could only be performed one time. As the aim was analyzing samples collected from the environment it would not be possible to repeat the experiment and provide exactly the same conditions while doing so.
Randomization	Observational study, no randomization necessary.
Blinding	Researchers were not blinded. During sampling it was not possible because samples were not divided into groups but were divided by sampling date. No potential sampling biases were anticipated as samples were not divided into treatment groups.

Did the study involve field work? ☐ Yes ☒ No

Reporting for specific materials, systems and methods

We require information from authors about some types of materials, experimental systems and methods used in many studies. Here, indicate whether each material, system or method listed is relevant to your study. If you are not sure if a list item applies to your research, read the appropriate section before selecting a response.

Materials & experimental systems

n/a	Involved in the study
☒	☐ Antibodies
☒	☐ Eukaryotic cell lines
☒	☐ Palaeontology and archaeology
☒	☐ Animals and other organisms
☒	☐ Human research participants
☒	☐ Clinical data
☒	☐ Dual use research of concern

Methods

n/a	Involved in the study
☒	☐ ChIP-seq
☐	☒ Flow cytometry
☒	☐ MRI-based neuroimaging

Flow Cytometry

Plots

Confirm that:

☒ The axis labels state the marker and fluorochrome used (e.g. CD4-FITC).

☒ The axis scales are clearly visible. Include numbers along axes only for bottom left plot of group (a 'group' is an analysis of identical markers).

☒ All plots are contour plots with outliers or pseudocolor plots.

☒ A numerical value for number of cells or percentage (with statistics) is provided.

Methodology

Sample preparation	In the current work, we used the last version of the flow cytometry Tara Oceans dataset published by Ibarbalz et al. 2019 Cell, 179, 1084-1097, which is open and publicly available and it was downloaded from http://dx.doi.org/10.17632/p9r9wttjkm.2 . Sampling, including preparation and processing is reported in Hingamp et al. 2013 ISME J 7.9: 1678-1695. Three aliquots of 1 ml of seawater (pre-filtered through 200-μm mesh) were collected from each depth. Samples were fixed immediately using cold 25% glutaraldehyde (final concentration 0.125%), left in the dark for 10min at room temperature, subsequently flash-frozen and kept in liquid nitrogen on board, and then stored at −80°C in the laboratory. Two sub-samples were taken for separate counts of heterotrophic prokaryotes and phototrophic picoplankton. For heterotrophic prokaryote determination, 400μl of sample was added to a diluted SYTO-13 (Molecular Probes Inc., Eugene, OR, USA) stock (10:1) at 2.5μmoll−1 final concentration, left for about 10min in the dark to complete the staining and run in the flow cytometer.
Instrument	FacsCalibur (Becton and Dickinson, Franklin Lakes, NJ, USA) flow cytometer equipped with a 15-mW Argon-ion laser (488nm emission).
Software	Paint-A-Gate software (Becton and Dickinson).
Cell population abundance	At least 30000 events were acquired for each subsample (usually 90000 events). Fluorescent beads (1μm, Fluoresbrite carboxylate microspheres, Polysciences Inc., Warrington, PA, USA) were added at a known density as internal standards. The bead standard concentration was determined by epifluorescence microscopy.
Gating strategy	Heterotrophic prokaryotes were detected by their signature in a plot of side scatter vs FL1 (green fluorescence). In a red (FL3) –green (FL1) fluorescence plot, beads fall in one line, heterotrophic prokaryotes in another and noise in a third (respectively, with more FL3 than FL1). Picocyanobacteria fall in between noise and heterotrophic prokaryote. This method is based on del Giorgio et al. 1996 Limmnol Oceanorgr 41: 783–789 as discussed in Gasol and del Giorgio (2000) Scientia Marina 64: 197–224. For phototrophic picoplankton, we used the same procedure as for heterotrophic prokaryote but without addition of SYTO-13. Small eukaryotic algae were identified in plots of side scatter vs FL3, and FL2 vs FL3 (Olson et al., 1993), and excluded in the enumeration of phototrophic prokaryotes.

☒ Tick this box to confirm that a figure exemplifying the gating strategy is provided in the Supplementary Information.
